# Changes in bone density and metabolism marker levels in type I Gaucher's disease following annual zoledronic acid hydrate administration: A case report

**DOI:** 10.1016/j.bonr.2026.101962

**Published:** 2026-10-20

**Authors:** Hiroyuki Onuma, Naoki Haraguchi

**Affiliations:** a Department of Orthopaedic Surgery, Kawasaki Municipal Tama Hospital by Designated Administrator Educational Foundation, St. Marianna University School of Medicine, Kawasaki, Kanagawa, 214-8525, Japan; b Department of Orthopaedic Surgery, St. Marianna University School of Medicine, Kawasaki, Kanagawa, 216-8511, Japan

**Keywords:** Gaucher's disease, Bone density, Bone metabolism, Bone resorption inhibitor

## Abstract

We present a rare case of type I GD with frequent bone complications. After multiple fractures the patient received 5 mg zoledronic acid hydrate, which improved TRACP-5b and P1NP levels. After 1 year, TRACP-5b levels increased significantly, and the patient received SRT therapy complemented with eldecalcitol for vitamin D deficiency. One year later, bone density had improved and bone metabolism markers remained within normal levels.

Osteoporosis treatment is a potential strategy for GD-related bone symptoms.

## 1 Introduction

Gaucher's disease (GD) is a rare genetic disorder with a global incidence ranging from 0.45 to 25.0 per 100,000 live births (Castillon et al., 2022). It is characterized by lipid accumulation in organs due to deficiency of the lysosomal enzyme glucocerebrosidase. GD is classified into three types according to the affected organs and symptoms: type 1 primarily affects the viscera, whereas types 2 and 3 are also associated with varying degrees of neurological impairment, either severe (type 2) or variable (type 3) (Stirnemann et al., 2017). Bone marrow infiltration by Gaucher cells can lead to mineralization defects caused by abnormal bone remodeling, osteoblast dysfunction, and inflammation-driven bone resorption (Hughes et al., 2019). Therefore, patients with GD frequently present with bone diseases and symptoms, such as osteopenia, leading to bone pain and weakening. This can result in bone deformities, pathological fractures, joint destruction, femoral head necrosis, and motor dysfunction, which significantly impact patients' quality of life (Hughes et al., 2019).

Current treatment options include enzyme replacement therapy (ERT) and glucosylceramide synthase inhibitors, which aim to prevent glucocerebroside accumulation (Kong et al., 2022). However, there is currently no cure for osteopenia in aging patients with GD (Cappellini et al., 2023) and treatments for primary osteoporosis, which aim to increase bone density and strength, may be beneficial.

Herein, we present a rare case of a patient with type I GD and frequent bone complications, in whom fractures and bone loss were successfully prevented with an annual administration of the bone resorption inhibitor zoledronic acid hydrate.

## 2 Case

### 2.1 Patient information

The patient was a 45-year-old male who presented with bilateral hip pain. He had a history of repeated fractures and decreased bone density on X-rays and had previously been diagnosed with GD.

The Bioethics Committee of St. Marianna University School of Medicine approved this study (approval number: 4913), and the patient provided verbal informed consent for the publication of this case report, including the use of X-ray and MRI images. The article does not contain any information that could reveal the identity of the patient.

### 2.2 Family history of GD and medical history

The patient had no family history of GD. His medical history revealed five previous fractures: one in the right elbow (6 years of age), two in the left lower leg (14 and 17 years of age), one in the left forearm (25 years of age), and one in the right shoulder (42 years of age). All of this information was obtained from the patient's records and through interviews with the patient.

### 2.3 Current medical history

The patient had experienced nosebleeds from the age of 4 years and was diagnosed with GD based on decreased β-glucosidase activity in cultured dermal fibroblasts. Regarding bone symptoms, the patient became aware of bone pain from the age of 5 years and had his first fracture at the age of 6 years. Subsequent fractures occurred at 14, 17, 25, and 42 years of age (Fig. 1). At 16 years old, the patient underwent splenectomy owing to increased abdominal distention. Although hemoglobin (Hb) and platelet (Plt) counts returned to within the normal range post-splenectomy, his bone pain persisted. The patient experienced hip joint pain at approximately 24 years of age and was diagnosed with bilateral femoral head necrosis based on magnetic resonance imaging (MRI) findings (images not available). MRI findings obtained at age 35 years are presented in Fig. 2. Following the fourth fracture, ERT with intravenous imiglucerase was initiated, which improved the patient's bone pain but not his hip joint pain. At 42 years old, the patient had a fifth fracture as a result of a fall. As the patient aged, preventing refracture and further crushing of the femoral head became a priority. At that time, the patient's lumbar spine (LS) bone mineral density (BMD), measured using DXA (Discovery, Hologic, USA, and Horizon Wi, Marlboro, Massachusetts, USA), was 0.766 g/cm^2^, with a young adult mean (YAM) value of 73%, indicating bone loss. Scoliosis and lumbar vertebral compression were observed on his X-ray (Fig. 3a and b, respectively). The acid phosphatase-5b level (tartrate-resistant acid phosphatase 5b; TRACP-5b) was 1.070 (reference range: 170–590) mU/dL, and the bone formation marker total procollagen 1 N-terminal propeptide (P1NP) was 101 μg/L (reference range: 18.1–74.1) (Fig. 4). All measured blood parameters were within the normal range (Table 1).

**Fig. 1:**
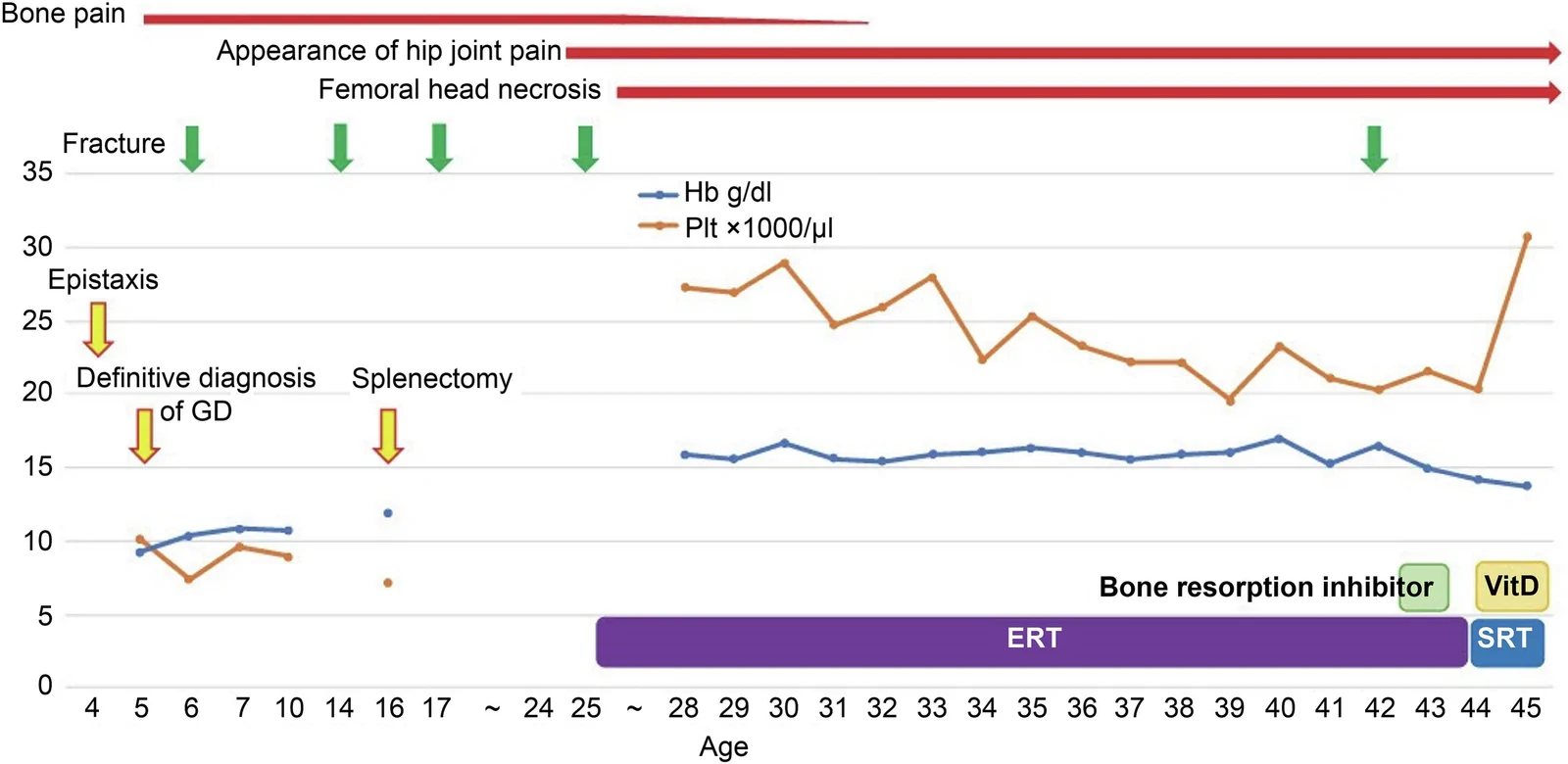
Clinical progress chart. Fluctuations in the patient's hemoglobin level and platelet count. Hb, hemoglobin; Plt, platelet; GD, Gaucher's disease.

**Fig. 2:**
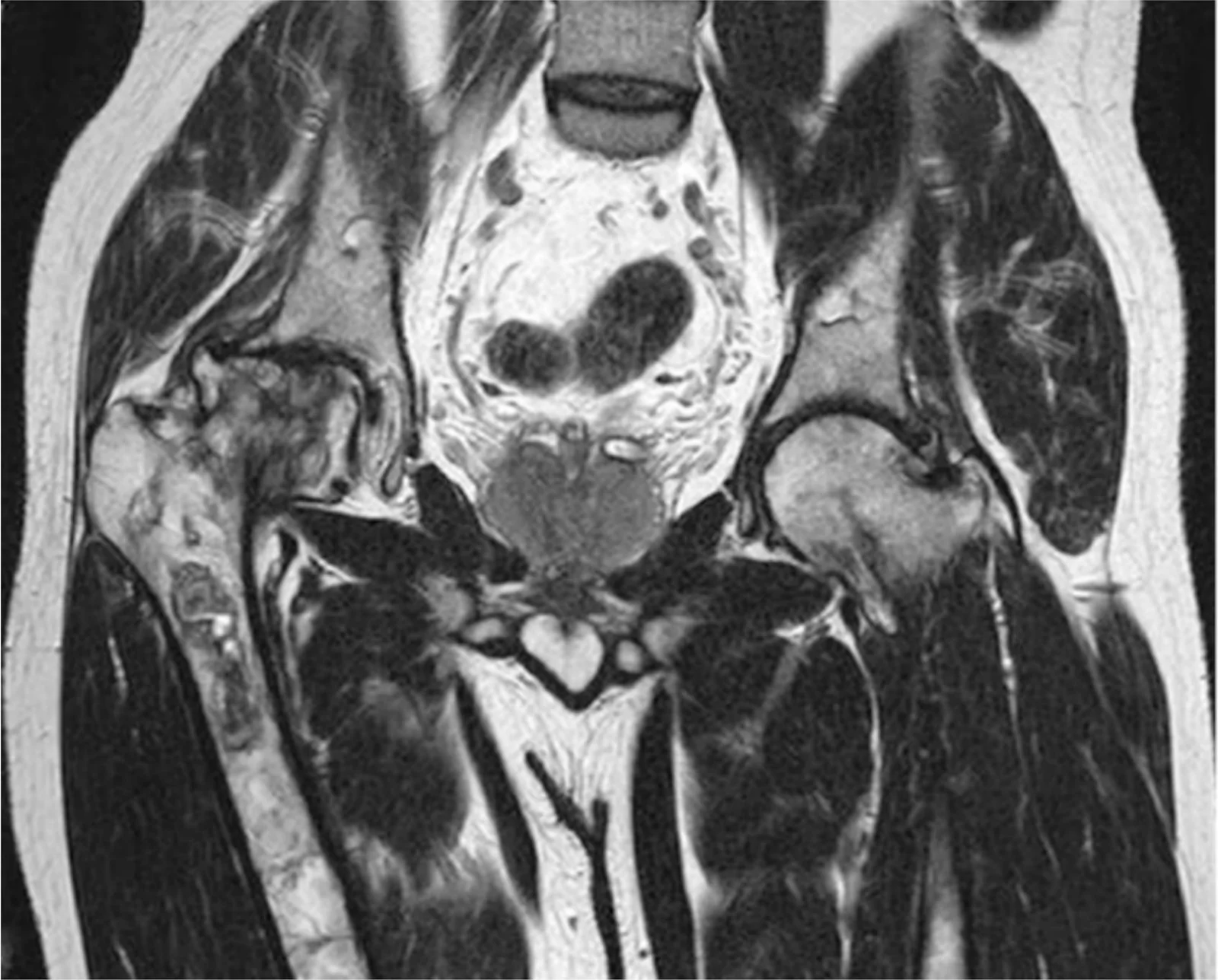
Hip joint, plain MRI (age 35) showing bilateral femoral head necrosis.

**Fig. 3:**
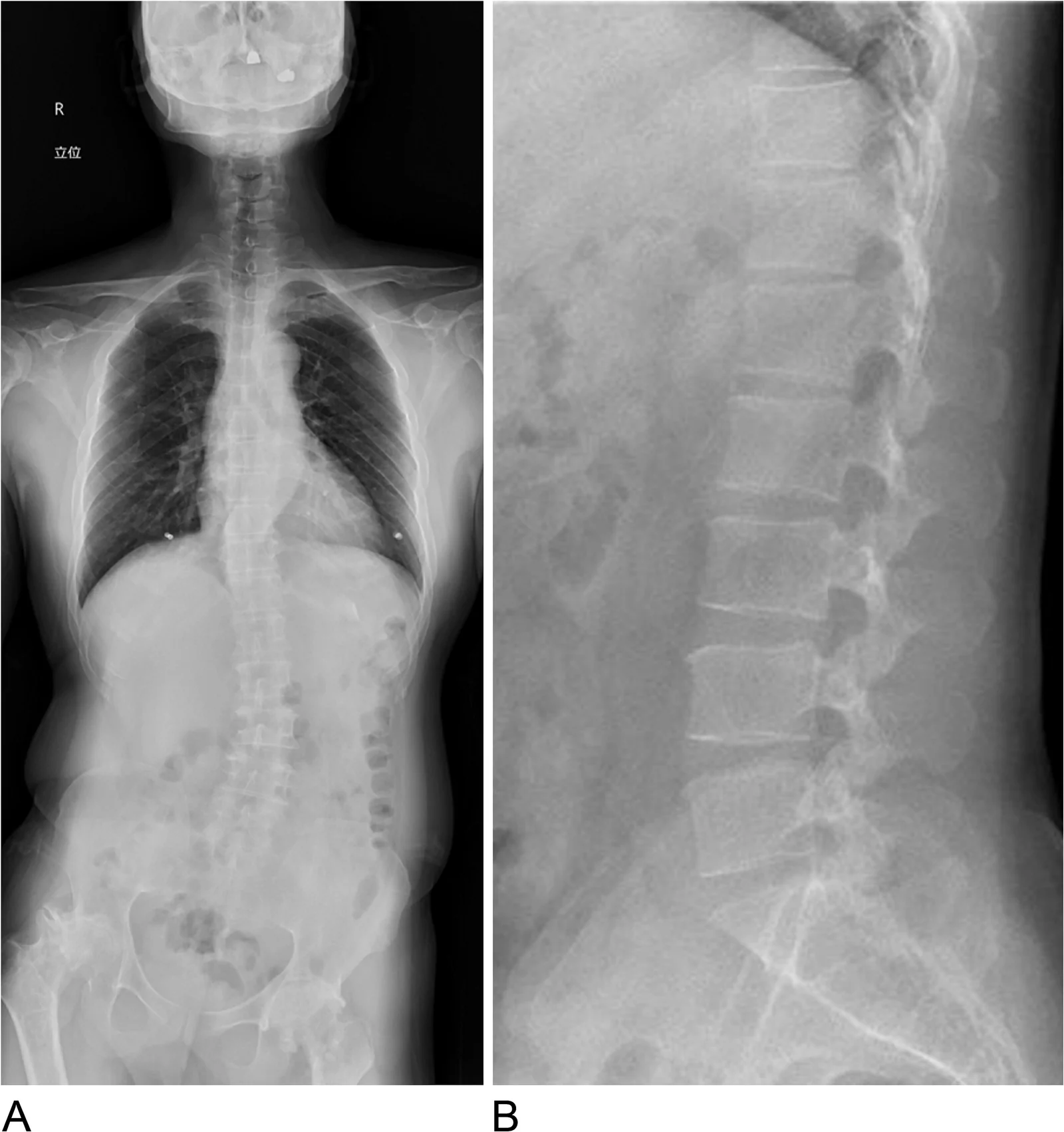
Whole spine, frontal view, plain X-ray (age 44). A) Scoliosis was observed in the spine; B) slight vertebral compression deformation was observed on plain lumbar spine X-ray.

**Fig. 4:**
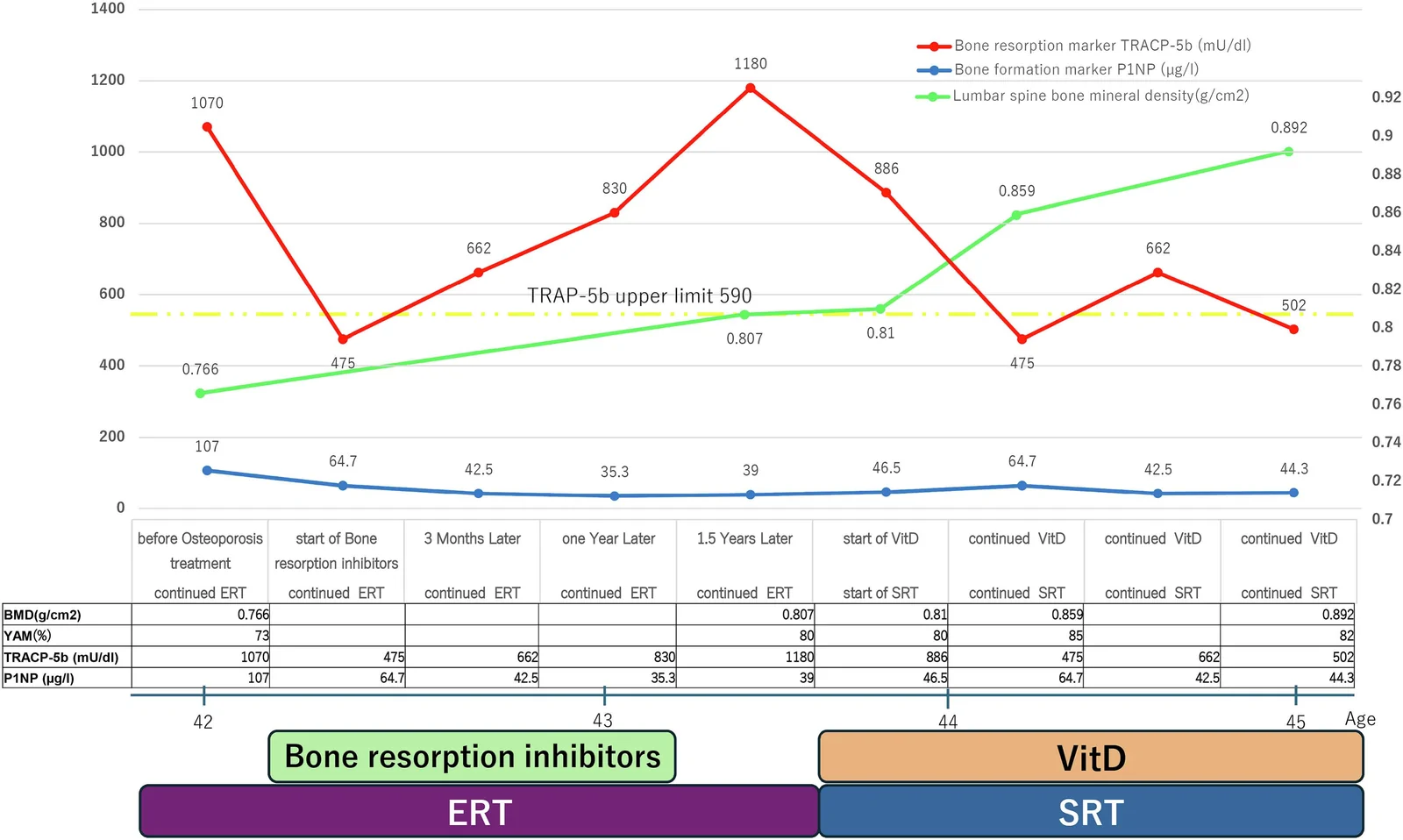
Fluctuations in bone metabolism marker levels and lumbar spine bone mineral density (LS-BMD). P1NP, procollagen 1 N-terminal propeptide; TRACP-5B, tartrate-resistant acid phosphatase 5b; BMD, bone mineral density; YAM, young adult mean; ERT, enzyme replacement therapy; SRT, substrate reduction therapy.

**Table 1:** Blood parameter levels measured during different treatment protocols.

Parameter	Reference range	01/09/17	11/10/17	08/11/17	13/12/17	24/03/18	16/04/18	22/11/18	03/06/19	16/12/19	17/02/20	20/08/20
Na (mEq/L)	138–146	NM	NM	141	NM	NM	141	139	142	141	141	142
CL (mEq/L)	99–108	NM	NM	105	NM	NM	106	103	105	105	104	103
K (mEq/L)	3.6–4.8	NM	NM	4.2	NM	NM	4.1	4.3	4.4	4	4.1	4.2
Ca (mg/dL)	8.6–10.2	10	8.9	9.4	9.9	10.3	9.3	9.4	9.4	9.3	9.3	10.3
P (mg/dL)	2.5–4.5	3.6	2.1	3.1	3.2	3.6	3.3	4.5	3.3	2.4	3.2	3.4
WBC (×10*2/μL)	36–93	NM	NM	64	56	NM	65	38	32	28	34	43
RBC (×10*4/μL)	431–565	NM	NM	520	509	NM	504	434	401	359	386	408
HGB (g/dL)	13.7–17.4	16.5	NM	16.9	16.5	NM	16.8	14.9	14.3	14.2	14.6	13.7
PLT (×10*4/μL)	15.7–38.2	20.3	NM	NM	NM	NM	19.3	21.6	19.7	20.3	21.0	30.8
AST (U/L)	11–32	20	17	23	19	26	22	21	22	25	21	22
ALT (U/L)	7–37	21	15	21	19	30	21	13	13	14	13	18
LD (U/L)	115–230	NM	NM	169	NM	NM	180	209	216	233	201	194
AL-P (U/L)	115–360	NM	NM	NM	NM	NM	207	190	194	211	176	192
γ-GTP (U/L)	5–72	NM	NM	35	NM	NM	31	23	19	21	21	42
CK (U/L)	40–200	NM	NM	NM	NM	NM	NM	87	98	158	107	199
TP (g/dL)	6.7–8.3	NM	NM	7.3	NM	NM	NM	7	7	6.7	7.1	7.1
Alb (g/dL)	3.9–4.9	4.3	3.9	4.4	4.3	4,6	NM	4.3	4.2	4.1	4,3	4.3
BUN (mg/dL)	8–22	NM	NM	12.2	16.5	NM	16.6	16.3	14.9	10.5	10.9	17.6
Cre (mg/dL)	0.61–1.04	0.82	0.65	0.76	0.75	0.78	0.78	0.74	0.88	0.81	0.87	1,2
eGFR (mL/min/1.73 m*2)	≧90	82.5	106.3	89.6	90.0	86.5	86.5	91.6	75.3	82.5	76.3	53.3
TRACP-5b (mU/dL)	170–590	830	475	NM	662	731	830	1180	886	724	NM	502
total P1NP (mg/L)	18.1–74.1	101	64.7	NM	42.5	42.5	35.3	39	46.5	38.6	NM	44.3

TP: total protein; NM: Not measured; the dashed vertical line represents the time point at which the treatment protocol changed from ERT to SRT + Vit. D.

The patient exhibited enhanced bone formation owing to significant bone resorption, as indicated by the P1NP level (Table 1, Fig. 4). Consequently, the bone resorption suppressant zoledronic acid hydrate was administered intravenously at a dose of 5 mg (Black et al., 2007; Nakamura et al., 2017), which decreased TRACP-5b and P1NP levels by 66% and 40%, respectively (Fig. 4). After 3 months of treatment, P1NP remained within the reference range (64.7 mg/L) whereas TRACP-5b had increased above the upper reference limit (662 mU/dL). After 1.5 years, the LS-BMD and YAM values had increased to 0.807 g/cm^2^ and 80%, respectively. However, the level of TRACP-5b had reverted to abnormally high levels, reaching twice the upper reference limit (1180 mU/dL). The YAM level remained at 80%, thus no additional dose of zoledronic acid hydrate was administered. At that time, the patient's 25-OH vitamin D level was 14.9 ng/mL, which was considered as deficiency (<20 ng/mL). Therefore, we began administering the active vitamin D preparation eldecalcitol (0.75 μg daily) and switched from ERT with imiglucerase to eliglustat substrate reduction therapy (SRT). One year later, the patient's LS-BMD value had increased to 0.892 g/cm^2^ and his YAM value was 82%, while both TRACP-5b and P1NP levels were within the reference ranges, as TRACP-5b had decreased by 57% compared with the level at the start of treatment (Fig. 4).

## 3 Discussion

Currently, there is no treatment for osteopenia in aging patients with GD. Herein, we present a rare case of a patient with GD who was treated with ERT after experiencing multiple fractures, femoral head necrosis, bone loss, and persistent hip pain. GD involves the accumulation of glycolipid glucosylceramide (glucocerebroside) due to reduced degradation as a result of deficiency of the enzyme β-glucocerebrosidase. Thus, the first approved treatment for GD was ERT with imiglucerase, which replaces β-glucocerebrosidase, thereby alleviating glucocerebroside accumulation. The second approved GD treatment was SRT with eliglustat, which inhibits glucocerebroside synthesis. Eliglustat has a shorter ineffective time than imiglucerase and increases BMD (Lukina et al., 2019).

In this case, the patient underwent ERT for 17 years from the age of 25 years and experienced bone loss as a consequence of enhanced bone resorption during remodeling. Although this process is part of normal bone metabolism, disordered remodeling disturbs the bone microenvironment, causing the cortex to become thinner and more fragile (Lecourt et al., 2012), which may lead to bone deformities such as Erlenmeyer flask deformation (Marcucci et al., 2014). These GD-specific bone symptoms affect patients' quality of life, particularly those with type I GD (Weinreb et al., 2007), leading to an increased need for supportive care.

The patient in this case had undergone splenectomy, which is an important risk factor for osteonecrosis (Mikosch and Hughes, 2010). ERT improved the patient's mean LS Z score, helping to avoid fragility fractures for the following 17 years (Sims et al., 2008). However, ERT alone did not maintain the peak level of normal adult bone mass. Even during ERT, the bone mass was further reduced owing to aging and to the 2-week administration interval. Therefore, to prevent further bone loss and improve bone density, the patient was evaluated and treated similarly to patients with primary osteoporosis. However, we believed that the treatment needed to be switched from ERT to SRT. Bone metabolism marker levels are crucial for evaluating bone lesions, guiding drug selection, and assessing the therapeutic effects of bone resorption inhibitors (Ivaska et al., 2010). Prior to administration of the bone resorption inhibitor, the patient had elevated levels of TRACP-5b (1070 mU/dL) and total P1NP (101 μg/L), indicating increased bone turnover. Although the YAM value in the present case was between 70% and 80%, the criteria for initiating osteoporosis treatment were met because there was a fragility fracture other than a proximal femur and vertebral body fracture. Therefore, zoledronic acid hydrate was administered, resulting in suppressed bone resorption. TRACP-5b and total P1NP levels normalized to 475 mU/dL and 64.7 μg/L, respectively. The LS-BMD and YAM values increased to 0.807 g/cm^2^ and 80%, respectively. This suggests that maintaining low TRACP-5b levels (within the normal range) (Komninaka et al., 2015) is important in patients with GD to prevent bone weakening. Zoledronic acid hydrate decreases TRACP-5b levels through its inhibitory effect on bone resorption. It is typically effective in inhibiting bone resorption for 1 year in patients with primary osteoporosis. However, in the present case, TRACP-5b levels increased again after 3 months. This finding is inconsistent with that of Nakamura et al. (2017), who observed that a similar dose produced sustained effects for 24 months in patients with osteoporosis. Therefore, we speculate that the effect of zoledronic acid hydrate might have decreased within a few months, suggesting that bone resorption may either be more intense in GD than in primary osteoporosis or involve a different mechanism. Furthermore, the selected dosage might not have been adequate to address GD symptoms; therefore adjusting the frequency and dose of zoledronic acid hydrate, similar to the management of denosumab (an anti-receptor activator of nuclear factor kappa-Β ligand [RANKL] antibody preparation) in rheumatoid arthritis (Takeuchi et al., 2019), might help maintain normal TRACP-5b levels and its bone resorption-suppressing effect.

Upon switching to eliglustat and eldecalcitol, the patient's bone resorption marker levels remained lower than those during ERT, and BMD increased with SRT treatment (Cox et al., 2017). SRT reduces osteoclast factor levels (Limgala and Goker-Alpan, 2020) and improves bone quality (Sidhu et al., 2020). Indeed, the patient's bone-related symptoms improved with SRT treatment. Furthermore, switching to denosumab may also be beneficial, given that bone destruction is mediated by osteoclasts and RANKL.

An additional observation in our patient was that he had a low blood concentration of 25-OH vitamin D (14.9 ng/mL), indicating vitamin D deficiency. This is consistent with the results of a previous survey of 74 patients with GD in the United Kingdom, which revealed a high prevalence of vitamin D deficiency (Mikosch et al., 2009). The administration of active vitamin D preparations can help prevent secondary osteoporosis, rickets, and osteomalacia (Saito et al., 2010) and can be initiated regardless of BMD. Vitamin D may improve bone symptoms in patients with GD as in patients with vitamin D-deficient rickets and osteomalacia. In this study, LS-BMD further increased to 0.892 g/cm^2^, highlighting the importance of combining SRT and osteoporosis treatment in managing osteopenia in patients with GD undergoing ERT.

Despite various interventions, the patient's hip joint deformities did not improve. This is consistent with a previous finding indicating that bone resorption inhibitors are most effective in early femoral head necrosis before crushing occurs (Lai et al., 2005). Therefore, to prevent a decrease in quality of life owing to bone complications in patients with GD, it is crucial to diagnose femoral head necrosis using MRI and initiate treatment before crushing is detectable via radiography, regardless of the presence or absence of symptoms. Femoral head necrosis often develops at a high rate following splenectomy in patients with GD. The sooner ERT is initiated, the lower the incidence of femoral head necrosis (Mistry et al., 2009). The diagnosis of GD is typically based on symptoms such as anemia, easy bleeding, and hepatosplenomegaly (Charrow et al., 2000), and approximately 50% of affected individuals are diagnosed by the age of 10 years. However, some patients with GD present only with mild symptoms, making timely diagnosis challenging.

Bone resorption inhibitors have been reported to improve bone density in patients with osteopenia (Wenstrup et al., 2004). Therefore, osteoporosis treatment is recommended for patients with GD. Additionally, similar to primary osteoporosis, lifestyle factors such as exercise, diet, and vitamin D intake should be considered in the management of bone symptoms of GD to prevent the deterioration of motor function, falls, and fractures.

Patients with GD undergoing long-term ERT presented with osteopenia and high levels of TRACP-5b and total P1NP (Ivanova et al., 2021). This case suggests that the annual administration of zoledronic acid hydrate may contribute to maintain normal levels of both bone metabolism markers for a short period but not over the long term, despite the observed BMD improvement, suggesting metabolic differences between GD and primary osteoporosis. However, this case has some limitations that should be considered when interpreting its findings. First, this is an uncontrolled, single-case report, which limits the generalizability of its findings. Second, there was an overlap of treatments, including zoledronic acid administration, a switch from ERT to SRT, and vitamin D repletion, complicating the attribution of the improvement in bone density to any single treatment. Therefore, the suggestion that a specific agent solely caused the increase in BMD should be avoided.

Further studies are needed to explore the efficacy of different bone resorption inhibitors, the effects of switching to SRT, and the administration of Vitamin D as adjuvant therapy. Moreover, given that the patient underwent several different treatments, caution should be taken when interpreting this case, as the outcome may have been influenced by the combined treatments.

Nevertheless, this case presents a possible therapeutic approach for patients with GD and osteopenia. Patients with GD and motor dysfunction often require supportive care, such as joint replacement and long-term care. Therefore, it is important to initiate treatment before significant bone destruction occurs.

## CRediT authorship contribution statement

**Hiroyuki Onuma:** Writing – original draft, Supervision, Methodology, Data curation, Conceptualization. **Naoki Haraguchi:** Writing – review & editing.

## Declaration of competing interest

We have nothing to declare.

## Data Availability

Data will be made available on request.
